# The Site Specific Demethylation in the 5′-Regulatory Area of NMDA Receptor 2B Subunit Gene Associated with CIE-Induced Up-Regulation of Transcription

**DOI:** 10.1371/journal.pone.0008798

**Published:** 2010-01-20

**Authors:** Mei Qiang, Ashley Denny, Jiguo Chen, Maharaj K. Ticku, Bo Yan, George Henderson

**Affiliations:** 1 Department of Pharmacology, The University of Texas Health Science Center at San Antonio, San Antonio, Texas, United States of America; 2 Department of Microbiology, The University of Texas Health Science Center at San Antonio, San Antonio, Texas, United States of America; 3 Department of Biological Sciences, Mississippi State University, Mississippi State, Mississippi, United States of America; 4 Department of Pharmacology and Neuroscience, School of Medicine, Texas Tech University Health Sciences Center, Lubbock, Texas, United States of America; National Institute on Drug Abuse, National Institutes of Health, United States of America

## Abstract

**Background:**

The NMDA receptor represents a particularly important site of ethanol action in the CNS. We recently reported that NMDA receptor 2B (NR2B) gene expression was persistently up-regulated following chronic intermittent ethanol (CIE) treatment. Increasing evidence that epigenetic mechanisms are involved in dynamic and long-lasting regulation of gene expression in multiple neuroadaptive processes prompted us to investigate the role of DNA methylation in mediating CIE-induced up-regulation of NR2B gene transcription. To dissect the changes of DNA methylation in the NR2B gene, we have screened a large number of CpG sites within its 5′-regulatory area following CIE treatment.

**Methods:**

Primary cortical cultured neurons were subjected to ethanol treatment in a CIE paradigm. Bisulfite conversion followed by pyrosequencing was used for quantitative measurement and analysis of CpG methylation status within the 5′-regulatory area of the NR2B gene; chromatin immunoprecipitation (ChIP) assay was used to examine DNA levels associated with methylation and transcription factor binding. Electrophoretic mobility shift assay (EMSA) and *in vitro* DNA methylation assays were performed to determine the direct impact of DNA methylation on the interaction between DNA and transcription factor and promoter activity.

**Results:**

Analysis of individual CpG methylation sites within the NR2B 5′regulatory area revealed three regions with clusters of site-specific CpG demethylation following CIE treatment and withdrawal. This was confirmed by ChIP showing similar decreases of methylated DNA in the same regions. The CIE-induced demethylation is characterized by being located near certain transcription factor binding sequences, AP-1 and CRE, and occurred during treatment as well as after ethanol withdrawal. Furthermore, the increase *in vitro* of methylated DNA decreased transcription factor binding activity and promoter activity. An additional ChIP assay indicated that the CIE-induced DNA demethylation is accompanied by increased occupation by transcription factors.

**Conclusions:**

These results suggest an important role of DNA demethylation in mediating CIE-induced NR2B gene up-regulation, thus implicating a novel molecular site of alcohol action.

## Introduction

Ethanol exposure on a chronic intermittent regimen has been found to produce behavioral excitability, seizure susceptibility, and increased anxiety [1], [2]. Repeated alcohol-withdrawal is known to induce long-lasting adaptations that underlie behavioral phenotypes associated with alcohol addiction. Although it is not understood mechanistically, addiction is thought to depend on molecular and cellular adaptations that lead to persistent changes in transcription, translation, and synaptic morphology [3], [4]. One such molecular neuroadaptation is up-regulation of glutamate transmission, an emerging target of ethanol action in the CNS. Glutamate is the primary excitatory neurotransmitter in the mammalian brain, especially in all cortical pyramidal neurons and thalamic relay neurons [5]. As a result, virtually all thalamocortical, corticocortical and corticofugal neurotransmission is mediated by glutamate [6]. Increases in *N*-methyl-D-aspartate receptor (NMDAR) subunit levels contributing to up-regulation of glutamate transmission by ethanol exposure were suggested by recent work showing up-regulated binding, function, and expression following chronic ethanol treatment [7]–[10]. Also, a mutation in the mouse NMDA subunit that alters the response of the channel to ethanol implies that the NMDA receptor is important in ethanol responses [11], [12]. Perhaps increased NMDAR function, which is considered to play a central role in the development of alcohol dependence, is at least partly due to an increase in expression of the NR2B subunit. Ifenprodil and eliprodil, two prototypes of NR2B subunit selective antagonists, potently inhibit ethanol withdrawal signs in alcohol-dependent animals [10]–[14]. Recent work in our laboratory and that of others has shown that CIE induces persistent up-regulation of NR2B gene expression and function [15], [16], [17].

Epigenetic mechanisms, including DNA methylation and histone modifications, change gene expression without alteration of the DNA sequence itself. DNA methylation, as one important epigenetic modification, was once thought to be a static process after cellular differentiation. However, it has recently been shown to regulate dynamically the adult nervous system in several important phenomena [18]–[30], including neurodegeneration and neuropsychiatric disorders [19], [27], [28], drug addiction [20], [21], plasticity and memory formation [22], [25], [26], [31] and activity-dependent regulation of neuronal activity [23], [29], [30]. A significant example is that the exon 1_7_ promoter of the glucocorticoid receptor in the hippocampus undergoes active demethylation which is independent of DNA replication as a response to maternal care after birth [24]. DNA methylation, particularly with its stable nature, offers an ideal mechanism to explain persistent cellular changes in neurons. This prompted us to determine whether DNA methylation serves as a mechanism involved in CIE-induced up-regulation of NR2B gene expression. If so, it may represent a novel mechanism for CIE-induced neuroadaptive regulation and, therefore, for the development of alcoholism related behavioral phenotypes. In the present study, we mapped for the first time changes in methylation levels in a large number of CpG sites in an alcohol target molecule, the NR2B gene. The result indicates both CIE treatment and its withdrawal induced a site-specific demethylation that in turn may lead to the up-regulation of the NR2B gene. This finding suggests a novel molecular site of ethanol action regarding NR2B gene transcription via an epigenetic mechanism.

## Results

### 5-Azacytidine (5′AZA) Induced Up-Regulation of NR2B Gene Transcription

**Figure 1:**
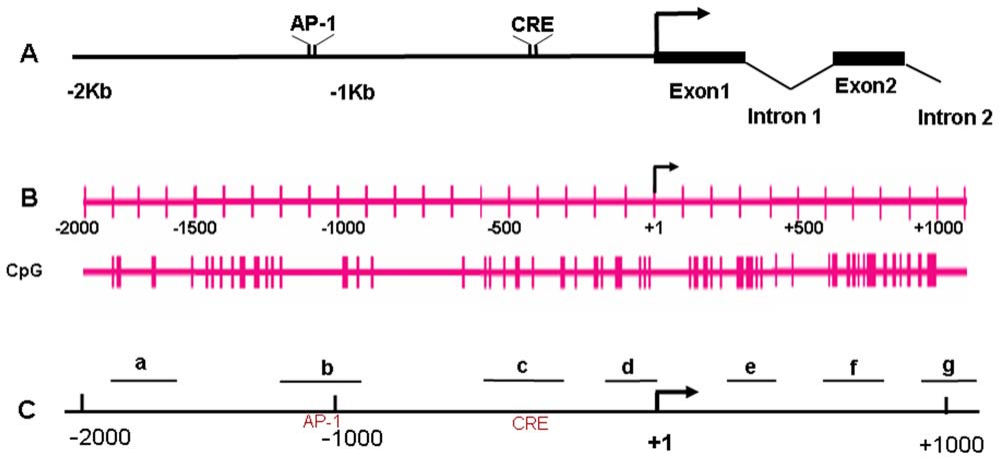
Schematic view of the 5′-regulatory area of the NR2B gene. **A**. The structure of 5′-regulatory area includes promoter (from −2000 bp to 0) and 5′-UTR region (from +1 to +1100 bp) of mouse NR2B gene, containing previously identified transcription factor CREB [38] and AP-1 [37] binding sites as indicated. **B**. The detailed distribution of CpG dinucleotides and their relative positions to AP-1 and CRE in 5′-regulatory area of the NR2B promoter is shown (note that the bars represent one or multi-CpG sites depending on the thickness). **C**. a to g represent the seven regions chosen in this study being amplified by qChIP PCR using different primer pairs in NR2B regulatory area and relative position in 5′-regulatory area of the NR2B (for the sequences of these primers see Table S1).

**Figure 2:**
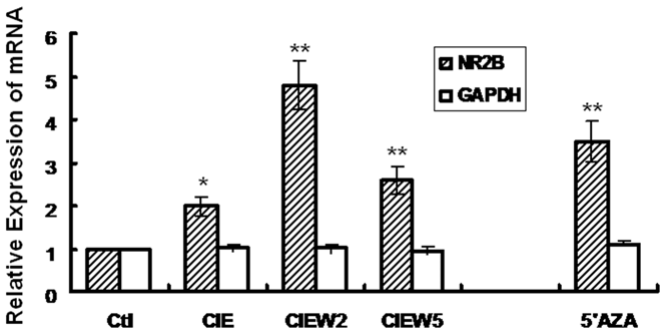
5′AZA induced up-regulation of NR2B gene expression. The cortical neuronal cultures were treated by ethanol in CIE regimen or 5′AZA (48 hrs), respectively. Total RNA was isolated and real time PCR for NR2B and GAPDH mRNA expression was performed. Results are presented as the mean ratio to control ± SEM; *, *p*<0.05; **, *p*<0.01 compared with control levels.

Our previous work demonstrated a significant CIE-induced up-regulation of NR2B gene transcription in cultured cortical neurons [15], [16]. Also an earlier study in our laboratory showed that continuous ethanol treatment caused a few of the CpG sites to be demethylated in the UTR in the NR2B gene [20]. We therefore set out to determine whether DNA methylation is involved in the CIE-induced phenomenon of NR2B gene transcription. The structure of the 5′-regulatory area of the NR2B gene was analyzed. As shown in Figure 1A&B, a CpG island with 116 CpG dinucleotides is located from upstream -2000 bp (promoter) to downstream +1100 bp (5′UTR) corresponding to the transcription start site. This suggests that this CpG-rich regulatory area might be potentially regulated by DNA methylation. To investigate this, 5′AZA, a DNA methyltransferase inhibitor which can cause extensive demethylation of 5-methyl-cytosine residues, was added to neuronal cultures at 5 µM for 48 hours. The results from real-time PCR analysis demonstrated that 5′AZA caused a significant increase in NR2B mRNA. In contrast, there was no such effect on the expression of the housekeeping gene GAPDH (Figure 2). To confirm that 5′AZA treatment indeed changes the DNA methylation in the 5′regulatory area of the NR2B gene, we treated neuronal cultures with 5′AZA for 48 hours and genomic DNA was isolated. Bisulfite-pyrosequencing, a newly developed sensitive and accurate method for the analysis and quantification of the degree of DNA methylation [32], was performed to examine methylation status in the individual CpG site. We found that 5′AZA causes an extensive demethylation (44% of observed CpG sites) in the NR2B gene after 48 h treatment (data not shown). This suggests that DNA methylation is involved in the regulation of NR2B gene; based on this, we hypothesized that the NR2B gene up-regulation caused by CIE and 5′AZA treatments may share the same mechanism, i.e. DNA demethylation.

### CIE Induced Site-Specific CpG Dinucleotides Demethylation

**Figure 3:**
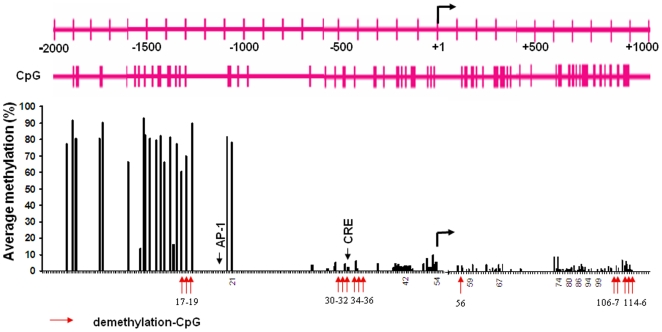
CIE induced DNA demethylation in the specific CpG sites determined by bisulfite-pyrosequencing. This graph outlines the relative CpG positions, where CIE induced significantly demethylation, to the transcription start site and the binding sites of AP-1 and CREB. Methylation status of individual CpG was determined by bisulfite-pyrosequencing at 116 CpG sites in the 5′-regulatory area of the NR2B gene. All bars represent the average basal methylation level (%) of individual CpG (control cells). The arrows in red only indicate these CpG sites where CIE induced significant demethylation (the detailed alterations are shown in Figure 4). The locations of transcription factor AP-1 and CREB binding sites are indicated by arrow. The bent arrow indicates the location of transcription start site.

**Figure 4:**
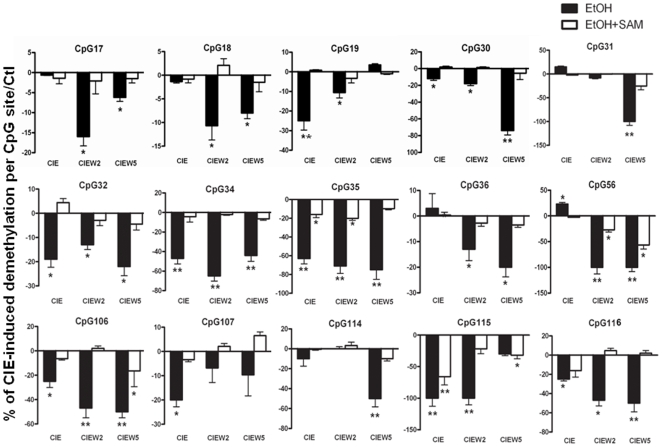
Quantitative analysis of DNA demethylation levels in the CpG sites following CIE treatment with or without SAM. Genomic DNA isolated from control and CIE-treated cortical neuronal cultures was bisulfite-treated and pyrosequencing analyzed. The 15 individual graphs show the detailed changes in percentage of CpG demethylation following CIE treatment and withdrawal with or without SAM in the indicated CpG sites, which are indicated with arrows in Figure 3. The values in negative are defined in this study to represent demethylation. Results are presented as the mean percentage of demethylation verse to the level of control (zero) ± SEM; *, *p*<0.05; **, *p*<0.01 compared with respective control levels.

To test this hypothesis, we investigated whether CIE treatment results in decreased methylation by using a bisulfite-pyrosequencing. Because it was unclear which CpG site may be sensitive to ethanol, we first established a methylation profile by scanning all 116 CpG sites across the area of 3kb NR2B promoter and 5′UTR. The cultured neurons were subjected to CIE treatment, and genomic DNA was isolated. Bisulfite conversion of genomic DNA was performed and PCR amplification was used with at least 20 primers spanning this area. The results from pyrosequencing were shown in Figure 3. We observed a basal pattern of methylation: a highly methylated region from −2.0 to −1.1 kb and a low methylated region from −1.1 to +1.1 kb. By mapping the changes of methylation in each individual CpG with regard to the CIE response, we found three chromatin regions with clusters of demethylated CpG sites, two of them in the promoter (CpG sites number 17–19 and 30–36, respectively) and one in the downstream second intron (CpG sites number 106–116). Figure 4 shows the detailed changes in demethylation levels in each of the 15 individual CpG sites after CIE treatment and withdrawal. Although the degree of CIE-induced demethylation varied from 10 to 100% compared to the control at these sites, there was a significant demethylation pattern after CIE treatment and withdrawal. Moreover, the demethylation in most of these CpG sites persisted after ethanol removal up to at least the fifth day of withdrawal, which is consistent with our previous finding of CIE-induced persistent up-regulation of NR2B gene transcription [15], [16].

**Figure 5:**
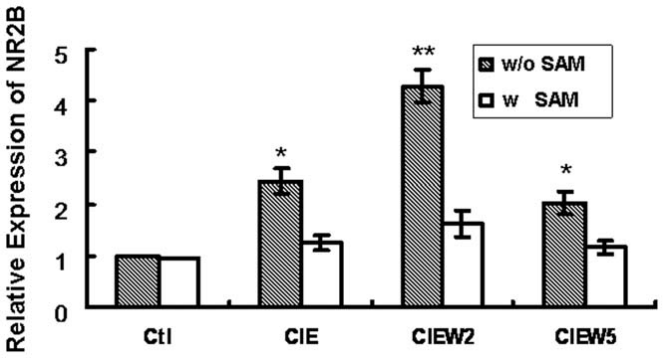
SAM prevents CIE-induced up-regulation of NR2B expression. The cortical neuronal cultures were treated by CIE regimen with and without the presence of SAM (2 mM). Total RNA was isolated and real time PCR for mRNA expression of NR2B was performed. Results are presented as the mean ratio to control ± SEM; *, *p*<0.05; **, *p*<0.01 compared with their corresponding control levels.

*S*-adenosyl-L-methionine (SAM) is the substrate for DNA methyltransferases (Dnmts) in methylation reaction, which has been previously used to rescue the hypomethylation [23]. To provide more direct evidence that DNA methylation contributes to CIE-induced up-regulation of NR2B gene expression, we added SAM to the cultured neurons during CIE treatment with expectation of blocking CIE-induced demethylation and, if this is the case, to prevent consequent increase of the NR2B mRNA level. The cells were harvested in the same way as that in the regular CIE paradigm. Bisulfite conversion and pyrosequencing were used to evaluate the methylation level; and RNA was isolated followed by qPCR analysis. As expected, the presence of excess SAM prevented both CIE-induced demethylation in the selected CpG sites (Figure 4) and consequent increase of NR2B gene mRNA compared to control (Figure 5).

The methylation data are not available for CpG sites #20–23 as we were unable to get our PCR primers to work at those sites. Attempts to amplify these DNA sequences by PCR using several redesigned primers and an alternative methylation specific PCR were also not successful. However, we have used other strategies to demonstrate the important role in these CpG sites (see results in ChIP assay). Taken together, these data indicate that DNA methylation in the 5′regulatory region of the NR2B gene is dynamically modified by CIE on selected site-specific CpG dinucleotides. Thus, CIE-induced DNA demethylation may contribute to the persistent up-regulation of NR2B gene transcription.

### CIE Decreased the Association of MeCP2 with Chromatin in the Corresponding Regulatory Regions of the NR2B Gene

**Figure 6:**
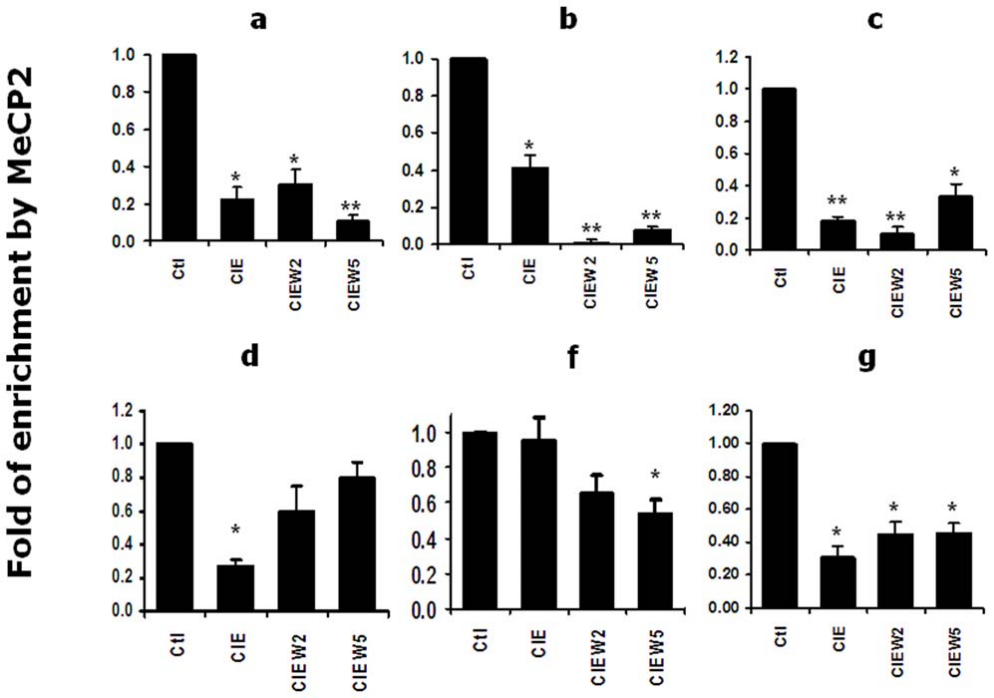
CIE reduced the association of MeCP2 with chromatin in specific regions. The chromatin fragments were prepared by cross-linking using 1% formaldehyde and precipitating with antibody specific to MeCP2. The qChIP PCR was performed to selectively amplify the region a, b, c, d, f and g. Each of the individual graphs represents the CIE-induced changes in region a to g (see Figure1). *, *p*<0.05; **, *p*<0.01 compared to the control.

To seek further evidence that CIE causes DNA demethylation in the specific regions of the NR2B gene, a series of primers were designed to amplify DNA fragments in 7 different regions (a through g in Figure 1C) across the NR2B 5′-regulatory sequences. MeCP2, reported to be an important component of the co-repressor complex [33]–[35], binds only to methylated CpG dinucleotides [36]. Therefore, a ChIP assay was performed using MeCP2 antibody (Abcam Inc., Cambridge) to pull down methylated DNA fragments. To test regionally specific changes within the 5′-regulatory area, the antibody-enriched methylated DNA was used as the template for qChIP PCR. The results show that MeCP2-enriched DNA fragments were significantly decreased in regions a, b, c and g, slightly decreased in d and f following CIE treatment and withdrawal (Figure 6), but not in region e (data not shown). This agrees with reduced DNA methylation activity by CIE in the corresponding chromatin regions, where demethylated CpG sites were identified. In addition, it is also consistent with CIE-induced persistent alterations in up-regulation of NR2B gene. However, the output, NR2B gene expression is a result of the multiple mechanisms, which reflects the complexity of the regulations. For example, the range of changes in methylation varies among these sites, some showing less demethylation on day 5 whereas others show more. With respect to MeCP2 occupancy, these data indicate that about half were at the same level at day 5 and at day 2 but others have started to increase (recover) by Day 5 compared with that in day 2.

### The Direct Role of DNA Demethylation on the Transcription Factor Binding and Promoter Activity

**Figure 7:**
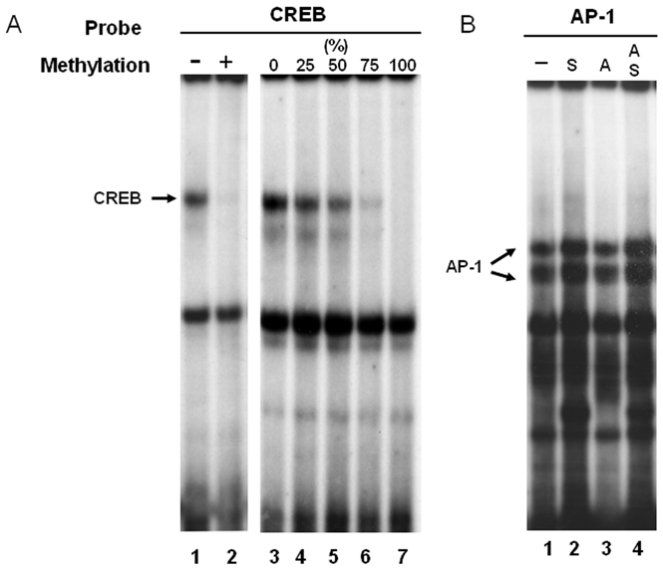
The influence of methylated CpG sites on the binding activities of CREB and AP-1. The representatives of four independent EMSA assays in each experiment are shown. **Left:** nuclear extracts from cortical neuronal culture were incubated with end-labeled double-stranded oligonucleotides with nonmethylated (Lane 1) and methylated (Lane 2) CpG 32 within CRE site. **Right:** nuclear extracts were incubated with probes incorporated with methylated CpG32 in 0%, 25%, 50%, 75% and 100% (Lane 3∼7). **2.** The probes of oligonucleotides containing AP-1 binding site from nonmethylated (Lane 1), hemi-methylated in sense (S, Lane 2) and anti-sense (A, Lane 3) and methylated (Lane 4) were used in EMSA. Since we have previously identified CREB [38] and AP-1 (family) [37] proteins binding by antibodies shift assay, the arrows indicate CREB or AP-1 bands.

**Figure 8:**
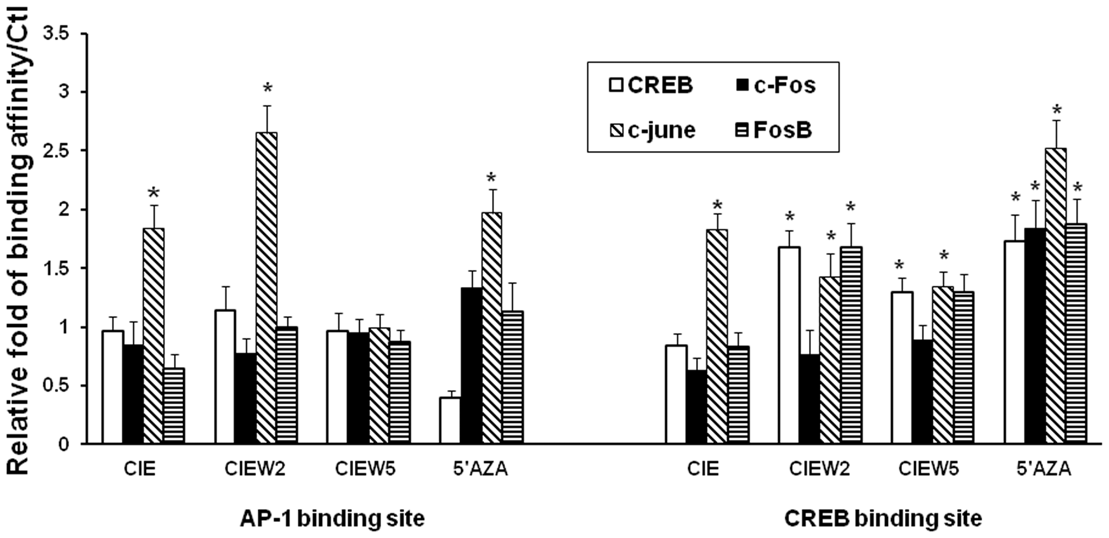
5′AZA and CIE cause increased the occupancy of transcription factors with chromatin. The cultured neuronal cells were treated by 5′AZA (4 days) or CIE. The chromatin fragments were prepared by cross-linking using 1% formaldehyde and precipitating with antibodies, CREB, c-Fos, c-Jun and FosB. The qChIP PCR was performed by using the primers amplifying the regions containing CRE and AP-1 sites, respectively. *, *p*<0.05 compared to the control.

Interestingly, region b and c contain transcription factors AP-1 and CREB binding sites, respectively. This suggests that CIE-induced DNA demethylation may influence NR2B expression by altering the binding of these transcription factors. Next, we investigated the mechanisms involved in demethylation mediated regulation of gene transcription by the NR2B gene promoter. The specific binding activities of AP-1 [37] and CRE [38] sites in the NR2B promoter have previously been identified by antibody shift assay. In this study, these two sites were chosen to determine whether methylation might directly interfere with transcription factor binding and promoter activity. EMSA was performed using synthesized oligonucleotides containing either non-methylated (C) or methylated cytosine (C^m^) within or near the transcription factor binding sites. We observed that binding activity in the CRE site is sensitive to DNA methylation at CpG 32. There, the presence of a methylated CpG inside the core sequence of CRE abolished the CREB binding. When different amounts of methylated DNA were incorporated, the binding activity was reduced proportionately *in vitro* (Figure 7A). The CIE-induced and demethylation agent, 5AZA-induced increases in transcription factors occupancy in this sites within the NR2B promoter were also confirmed by ChIP Assay (Figure 8). The results suggest that the methylation status of a specific CpG site (CRE) could alter the accessibility of its transcription factor to this site. In contrast, the binding activities of the oligos containing two CpG flanking the AP-1 site were not reduced by methylation in EMSA (Figure 7B). However, ChIP array showed CIE and 5′AZA induced an increased binding activity of c-Jun, implicating the existence of indirect mechanism.

**Figure 9:**
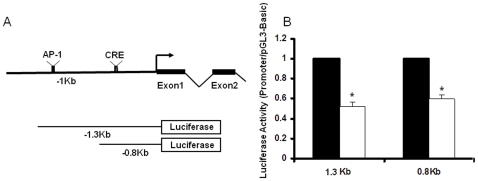
*In vitro* methylation inhibits promoter activities. A. The structure of the luciferase reporter gene constructs of the NR2B gene promoter, containing CREB (0.8 kb) or both CREB and AP-1 (1.3 kb) binding sites. **B**. Constructs 1.3 and 0.8 kb were *in vitro* mock-methylated and methylated and transfected into cultured cortical neurons. Cells were collected 48 h after transfection. Solid and open bars represent the luciferase activities from mock-methylated and methylated promoter, respectively. Data (mean ± SEM) shown are from at least three independent experiments. *, *p*<0.05 compared to the mock-methylated control constructs.

To determine whether methylation state and the corresponding changes in CREB binding activity contribute to the functional difference in transcription of NR2B gene, two DNA fragments in NR2B promoter containing AP-1 and CRE sites (−1.3k∼+30) or CRE site (−800∼+30) only were *in vitro* methylated in the CpG sites and then cloned into luciferase reporter vector (Figure 9A). These vectors were transfected into neuronal cultures, and dual luciferase assay was performed. The results from the *in vitro* methylation assay showed that the CREB binding site was sensitive to methylation, i.e. methylation of the fragment containing the CRE element in the NR2B promoter reduced promoter activities by 60%. However, and methylation had no apparent effect on AP-1 driven promoter activity (Figure 9B).

In summary, the demethylation of NR2B gene promoter might increased CREB binding to the promoter and activated transcriptional activity of the NR2B gene. This suggests a mechanism that mediates CIE-induced up-regulation of NR2B gene transcription. On the other hand, the mechanism by which the AP-1 binding site in the NR2B gene mediates ethanol effects on transcription, as previously reported by others, may involve indirect effects of CpG demethylation.

### CIE Induced Decrease of Dnmt1 Levels

**Figure 10:**
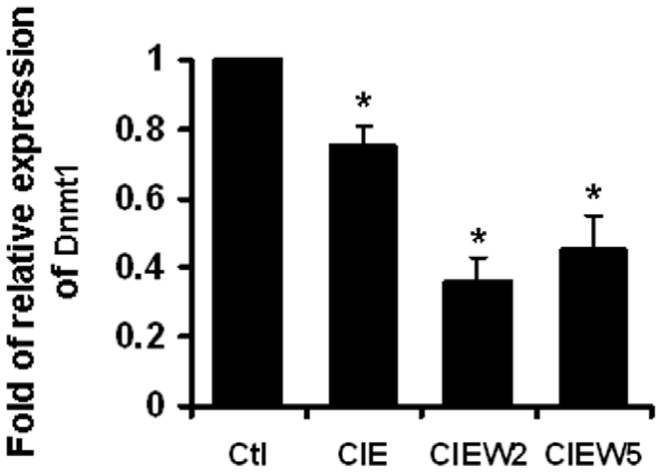
CIE reduced Dnmt1 expression. The cortical neuronal cultures were treated by CIE regimen. Total RNA was isolated and real time PCR was performed. Results are presented as the mean relative ratio to control ± SEM; *, *p*<0.05 compared with control level.

DNA methylation is catalyzed by the Dnmts [39]. Dnmt1 has been reported as a crucial regulator in DNA methylation in the genes responsible for the functions in brain (40–42). Therefore, it is possible that Dnmt1 mediates CIE induces DNA demethylation. Therefore, Dnmt1 mRNA content was examined by real time PCR. As shown in Figure 10, CIE induced a decrease in the level of Dnmt1 mRNA with a lasting effect.

## Discussion

Recent studies have demonstrated that DNA methylation is a dynamically regulatory process in the adult nervous system [18]–[30]. An emerging concept is that the regulation of chromatin structure through DNA methylation and histone acetylation may mediate long-lasting changes in complicated behaviors [21], [22], [29], [43]. However, how methylation changes these behaviors by altering gene expression is still unknown. NMDARs are one of the main targets of ethanol action in the brain and, as such, have been implicated in mediating ethanol-associated phenotypes such as intoxication, tolerance, dependence, withdrawal, craving, and relapse [44]. Consequently, NMDA receptors may also be critical targets for epigenetic regulation in alcohol addiction. In this study, we have identified a molecular target of epigenetic regulation in alcohol addiction by focusing on the role of CIE-induced changes in DNA methylation within the 5′-regulatory area of NR2B gene. Our data reveal three clusters of CpG dinucleotides in which CIE treatment induced a significant decrease of DNA methylation levels. The phenomenon of CIE-induced demethylation was characterized by regional specificity and persistence following ethanol removal. This provides strong support for our hypothesis that DNA methylation mediates CIE-induced persistent up-regulation of NR2B gene transcription. The DNA methylation pattern is believed to be a relative balance of DNA methylation and demethylation. To achieve the regulation to mature nervous system, DNA methylation would also have to be an active and reversible process to enable neurons to respond to physiological and environmental stimuli. However, whether there is active DNA demethylation in postmitotic cells, such as neurons, is one of the longstanding controversies. The relevant questions also include how the decrease of Dnmt1 results in DNA demethylation in postmitotic neurons. There is increasing evidence recently suggesting active methylation and demethylation regulation in mediating diverse changes in the adult CNS [19], [20], [23], [24], [29], [31], [45]–[51], which is challenging the common accepted mechanisms [for review see 4], [52]. There are also reported studies showed that Dnmt1 plays a critical role in dynamic DNA methylation and regulation of gene expression in the adult mammalian nervous system [24], [31], [41], [42], [49]; administration of 5′AZA is able to significantly inhibit DNA methylation in postmitotic neuronal cells or adult brain [53], [54]. The present data agree with these recent findings and add new evidence for the active demethylation regulation in the mechanism of ethanol addiction.

The commonly accepted mechanism of 5′AZA action is based on the initial report [55] that incorporation of cytidine analogs into DNA in replicating cells inhibits the ability of these enzymes to methylate DNA. However, it has been reported that administration of 5′AZA is able to significantly inhibit DNA methylation in postmitotic neuronal cells or adult brain [53], [54]. Furthermore, the idea that 5′AZA inhibits methylation through a tight covalent complex between Dnmts and 5′AZA-substituted DNA has also been challenged. DNA methyltransferase activity decreases much faster than incorporation of 5′AZA into DNA [56]. The 5′AZA-induced alteration in expression occurs independently of the growth stage and is not due solely to the incorporation of the analog into DNA [57]. It was also found recently that 5′-AZA-Deoxycytidine induces a proteosomal degradation of DNMT1 that starts earlier than the incorporation of 5′AZA into DNA [58]. Thus, there are alternate mechanisms by which 5′AZA can cause demethylation other than by its incorporation into DNA during cell division.

In the present study, we also use SAM, a methyl donor, to specify demethylation involved in CIE-induced up-regulation of the NR2B gene. We found that it can prevent CIE-induced up-regulation of NR2B gene expression through blocking the induction of demethylation. The mechanism by which SAM increases DNA methylation has been suggested to be either by stimulating DNA methylation enzymes [59] or by inhibiting demethylases [60] although it need to be confirmed in the neuronal cells.

DNA methylation results in repression of gene transcription and loss of methylation are associated with activation of transcription activity [61] by altering chromatin configuration and the accessibility of transcription factor [62], [63]. By focusing the affect of methylation on transcription activity, we looked into the relationships between the DNA methylation status of the NR2B promoter and transcription factor accessibility as well as gene expression. We found that two out of the three clusters of demethylated CpG dinucleotides coincide with consensus AP-1 and CREB binding sites. These two transcription factors have previously been identified to regulate NR2B expression in response to chronic ethanol treatment [37], [38], therefore, the regionally specific demethylation may be critical in regulating promoter activities mediated by AP-1 and CREB. To address whether methylated CpG in a particular site interferes with the binding of these transcription factors, we first examined the interfering on binding activities of these two transcription factors by EMSA. A methylated CpG within the CRE recognition sequence blocked CREB binding. Conversely, loss of methylation with the CRE recognition was associated with recovery of the affinity binding that was proportional to methylated CpG. This finding confirmed the direct role of DNA methylation in altering the accessibility of transcription factors to the NR2B promoter. This result was also confirmed by ChIP assay and qPCR. By these analyses, we showed that 5′AZA-induced demethylation was accompanied with the increase in the interactions between CREB, c-Fos, c-Jun and FosB and the region containing CRE element, which is similar with CIE-induced increases of the occupancy of the NR2B promoter with CREB and c-Jun. Furthermore, we further investigated the functional importance of methylation status for altering gene expression in this site by *in vitro* methylation of the region surrounding this binding site. Thus, increased methylation resulted in inhibition of promoter activity. Together, these data show that site-specific DNA demethylation in NR2B mediates CIE-induced up-regulation of NR2B gene transcription. However, this may not hold true for other regions. For example, we found no similar change in the AP-1 site as indicated by the lack of change in transcription factor binding and promoter activity. One possible explanation might be that AP-1 regulates NR2B transcription through a more complicated chromatin remodeling mechanism, e.g. histone modifications, rather than the direct effect of demethylation in these neighboring CpG sites. DNA methylation and histone modifications are critical epigenetic processes controlling chromatin structure and gene regulation [64], [65]. Recent studies have suggested a complicated interaction between them [66]–[68]. Thus, perhaps DNA demethylation and histone acetylation work in concert at the AP-1 site to regulate CIE-induced NR2B gene up-regulation. Further studies are required to determine the precise regulation in this process.

Although we initially used MeCP2 antibody in ChIP assay to identify methylated DNA fragment based on the earlier published studies [33]–[35], we were surprise to find that CIE-induced demethylation was accompanied by reduced occupancy of MeCP2 in the NR2B promoter and by an increase of NR2B gene expression. We also found that knockdown of MeCP2 with lentiviral shRNA in cultured cortical neurons results in a significant increase of NR2B mRNA (data not shown), which is consistent with previous reports that MeCP2 serves as a transcriptional repressor [33]–[35]. However, several studies also suggested an opposite role of MeCP2 on regulating a diversity of genes as a transcriptional activator [69]–[71]. A more recent study revealed that MeCP2 regulates the expression of a wide range of genes in the hypothalamus as both an activator and a repressor of transcription [71]. A possible explanation is that MeCP2-involved regulations of gene expression might differ in specific brain regions or type of neurons. The further study is required in addressing this issue.

Although the mechanism of how CIE induced DNA demethylation is unknown, the role of two enzymes, Dnmts and demethylases is the important focus. Emerging evidence suggests the existence of demethylse [46], [72], especially in postmitotic cells [24], [31], [48], including the most recent study in Gadd45b mediated activity-induced DNA demethylation in specific promoters and gene expression for adult neurogenesis [29]. Also, the role of Dnmt1 is implicated in the postmitotic neuronal cells, since Dnmt1 is not only highly expressed in dividing cells but is also abundant in adult brain [40]–[42]. The function of Dnmt1 was commonly accepted to be responsible for maintaining DNA methylation patterns in the daughter strands of dividing cells and in development of brain. So, the only way methyl groups were thought to be lost was through passive demethylation (i.e. replication in the absence of DNA methyltransferase) and such processes were not considered applicable to postmitotic neurons [73]. However, accumulating evidence has revealed that Dnmt1 plays a critical role in dynamic DNA methylation and regulation of gene expression in the adult mammalian nervous system [24], [31], [41], [42] [49]. For example, a recent study showed that expression of Dnmt1 mRNA is increased in prefrontal cortical GABAergic neurons of schizophrenia patients with decreased GAD67 level [42]; while nicotine injections for 4 days decreased Dnmt1 mRNA and protein with increased GAD67 expression in the mouse frontal cortex [49]. The reduction of Dnmt1 through shRNA or antisense oligonucleotides knockdown, as well as using DNA methyltransferase inhibitor results DNA demethylation in specific genes [31], [50], [51]. We also found that CIE-treated neuronal cultures resulted in lasting reduction in the level of Dnmt1, which suggests that Dnmt1 is involved in CIE-induced NR2B demethylation although the exact mechanism needs to be addressed in the future study.

Studies of human addiction and behavioral studies in rodent models of addiction have indicated that key behavioral abnormalities associated with addiction are extremely long lived [3]. It is particularly interesting because one critical aspect of alcohol dependence is the lasting alterations in gene expressions during withdrawal adaptations. Persistent up-regulation of NR2B gene expression by chronic intermittent alcohol treatment may serve as one mechanism underlying ethanol dependence behaviors. The present data showed CIE-induced demethylation during a five-day-withdrawal. Although its contribution to the ethanol related behaviors has yet to be investigated in an animal model of alcohol addiction, the finding in this study suggests the potential mechanisms of epigenetic regulations in alcohol dependence. Thereby, it might be an important focus of pharmacologic approaches.

## Materials and Methods

### Cell Culture and CIE Model

Primary cortical neurons were prepared from C57BL/6 mouse fetuses as described previously [74] with minor modifications. The cultured cells were assigned to one of the four groups: Control (Ctl), neurons were kept in normal medium and subjected to the media change as other groups; CIE, neurons were exposed to 75 mM ethanol for 5 cycles, each cycle consisting of 14 h ethanol exposure followed by a 10 h intermittent withdrawal (neurons were harvested in the last cycle after 14 h ethanol treatment); CIEW2, CIE plus 2 days of full withdrawal; and CIEW5, CIE plus 5 days of full withdrawal. The ethanol-treated neuronal cultures were kept in an incubator saturated with ethanol, which maintained the ethanol concentration at the level added to the medium as determined by monitoring alcohol concentration using an Analox AM1 analyzer. During withdrawal cycles, cultures were kept in a separate ethanol-free incubator. In view of the developmental changes during culture, we started treatments for the different groups at different times and harvested cells at the same age of cultured neurons (e.g., Ctl, no ethanol treatment; CIE, starting ethanol treatment on DIV 8; CIEW2, on DIV 6; and CIEW5, on DIV 3).

### Bisulfite Conversion and Pyrosequencing

Genomic DNA (1 µg) isolated from cortical neuronal cultures using a Blood and Cell Culture DNA kit (Qiagen), was bisulfite-treated using the Zymo DNA Methylation Kit (Zymo research, Orange). Bisulfite-treated DNA was eluted in 10 µl volumes with 1 µl used for each PCR. PCR was performed with primers biotinylated to convert the PCR product to single-stranded DNA templates. The PCR products (10 µl) were sequenced by pyrosequencing using the PSQ96 HS System (Biotage, Kungsgatan, Sweden) following the manufacturer's instructions. The methylation status of each locus was analyzed individually as a T/C SNP using QCpG software [75].

### Real-Time PCR

Using 1 µg of total RNA as template, single-stranded cDNAs were synthesized using random hexamers and TaqMan reverse transcription reagent kit (Applied Biosystems, Branchburg). For the real time PCR reaction, specific probes (Applied Biosystems) were used for NR2B expression and the SyBr green plus primers were used for other examinations. Real-time PCR was performed using the ABI Prism 7900 sequence detection system. The 2^−ΔΔCt^ method was used for quantification with 18s or GAPDH as endogenous controls. Gene expression in CIE-treated cells was expressed as fold changes by comparing to that in control cells.

### Chromatin Immunoprecipitation (ChIP) Assay and Quantitative PCR (qChIP PCR)

ChIP assays were performed as previously described [37] by using a ChIP Express kit (Active Motif, Carlsbad). Briefly, primary cultured cortical neurons were cross-linked with 1% formaldehyde for 10 min at room temperature, then glycine was added to stop the fixation, cell pellets were lysed, and nuclei were collected. The lysed extracts were subjected to shearing by sonication. One-tenth of the sheared DNA was kept to evaluate fragment size and DNA concentration before immunoprecipitation and used as input. The sizes of the majority of sonicated genomic DNA ranged from 300 to 600 bp. Equal amounts of soluble chromatin from each sample were subjected to immunoprecipitation with different antibodies or IgG (negative control) as indicated. The complexes were drawn off with protein G magnetic beads and washed sequentially with low-salt, high-salt, LiCl, and Tris-EDTA buffers. The cross-links were reversed and followed by proteinase-K treatment. DNA fragments were then purified with a Qiagen DNA extraction kit.

Levels of specific methylation, histone acetylation and transcription factor binding in the 5′regulatory area of the NR2B gene were determined by measuring the amount of antibody enriched chromatin DNA by quantitative real-time PCR. Primers were designed to amplify specific regions in the 5′-regulatory area of the gene.

Controls were included to confirm specificity and validity. To control the specificity of antibody binding, we used non-immune rabbit IgG (Santa Cruz Biotechnology) to immunoprecipitate chromatin samples, which resulted in negligible levels of real time PCR activity (data not shown). For internal control of qChIP PCR, we designed the primers to amplify GAPDH promoter (F: 5′-ccaaagacagaagccaggag-3′; R: 5′-catcgaacctctccccatta-3′). The levels of DNA associated with methyl-DNA binding proteins and transcription factors at the promoter of GAPDH gene were measured; no difference was found after CIE-treat treatment (data not shown).

### Electrophoretic Mobility Shift Assay

Nuclear extracts from cultured cortical neurons were prepared as previously described [74]. Complementary pairs of site-specific methylated (C^m^) and non-methylated (C) oligonucleotides (Sigma-genosys) from the regions of AP-1 and CRE in the NR2B promoter were prepared and annealed. AP-1: 5′-AGC TGG GTT A**GT CA**G TGC TGT AAA ATC ATT TC(C/C^m^)G TAT GGG AAA CAT TC(C/C^m^)G TTT-3′. CRE: 5′-CCC CAG CTC A**TG AC** (C/C^m^)**G** **TGA** GAA GCA GCC TCA-3′. Sequences in bold represent the core sequences of the NR2B gene promoter AP-1 and CRE sites; two adjacent methylated CpGs near the AP-1 site and one methylated CpG inside the CRE site are underlined. The double-stranded oligo nucleotides were end-labeled with [γ-^32^P]ATP. For reaction, 12 µg of nuclear extracts were used. After reaction, the samples were separated by 5% non-denaturing polyacrylamide gels, and electrophoresis was carried out. The gels were dried subsequent to autoradiography.

### In Vitro DNA Methylation and Luciferase Assay

The DNA fragments from mouse NR2B promoter regions ranging from −1300 to +30 bp and from −800 to +30 bp were methylated with bacterial CpG methylase SssI or mock-methylated *in vitro* as described elsewhere [76]. These methylated DNA fragments were then inserted into luciferase reporter gene vector pGL3-Basic. Primary cultured cortical neurons grown in 24-well plates were transfected with these vectors on DIV 5 by lipofactamine transfection kit (Invitrogen). To control transfection efficiency, a *Renilla reniformis* luciferase vector phRG (Promega) was added per well. Cells were harvested 48 h after transfection for luciferase activity assayed in a Turner Designs TD-20/20 luminometer using the dual luciferase assay system according to manufacturer's protocol (Promega).

### Statistics

Two-way ANOVA followed by Newman-Keuls multiple comparison tests were used to assess the significance of the differences between CIE treatments and individual CpG methylation levels in bisulfite-pyrosequencing data. One-way ANOVA followed by Newman-Keuls multiple comparison tests were used for all other data when necessary.

## Supporting Information

Table S1 (0.03 MB DOC)
