# Supplementary material for: The Site Specific Demethylation in the 5′-Regulatory Area of NMDA Receptor 2B Subunit Gene Associated with CIE-Induced Up-Regulation of Transcription
Source: PLoS One. 2010 Jan 20;5(1):e8798. doi: 10.1371/journal.pone.0008798 (PMC2808353; doi:10.1371/journal.pone.0008798)
Supplement: Table S1 — (0.03 MB DOC) [file pone.0008798.s001.doc]

**Table S1**

________________________________________________

Regions Primers Sequences (5’~3’)

________________________________________________

a Forward gtttgggcaacaggagaaat

Reverse gtgactggaacaaaggcaga

b Forward agggagagaaatggctgcta

Reverse gctgggaaagatttgaggac

c Forward caggggagtggtttcagtgt

Reverse ccctcactcccactgctaag

d Forward gacccaaatcaagaccagga

Reverse caagagagcccagattccag

e Forward gatccatttatcctgctttgc

Reverse tgatttctccaaaccctcaa

f Forward acaggactgcctttggtctt

Reverse tcaatgggttctgattgtgc

g Forward gctggaggagatgaggagag

Reverse aagtggaaagcaaggaggaa

_________________________________________________
